# The Gateway to the Brain: Dissecting the Primate Eye

**DOI:** 10.3791/1261

**Published:** 2009-05-27

**Authors:** Mark Burke, Shahin Zangenehpour, Joseph Bouskila, Denis Boire, Maurice Ptito

**Affiliations:** Department of Physiology, Universite de Montreal - University of Montreal; School of Optometry, Universite de Montreal - University of Montreal; Departement de chimie-biologie, Universite du Quebec a Trois-Rivieres

## Abstract

The visual system in humans is considered the  gateway  to the world and plays a principal role in the plethora of sensory, perceptual and cognitive processes. It is therefore not surprising that  quality of vision  is tied to  quality of life . Despite widespread clinical and basic research surrounding the causes of visual disorders, many forms of visual impairments, such as retinitis pigmentosa and macular degeneration, lack effective treatments. Non-human primates have the closest general features of eye development to that of humans. Not only do they have a similar vascular anatomy, but amongst other mammals, primates have the unique characteristic of having a region in the temporal retina specialized for high visual acuity, the fovea^1^. Here we describe a general technique for dissecting the primate retina to provide tissue for retinal histology, immunohistochemistry, laser capture microdissection, as well as light and electron microscopy. With the extended use of the non-human primate as a translational model, our hope is that improved understanding of the retina will provide insights into effective approaches towards attenuating or reversing the negative impact of visual disorders on the quality of life of affected individuals.

**Figure:**
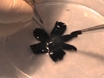


## Protocol

### Part 1: Pre-processing of tissue

Tissue should be well perfused with paraformaldehyde, glutaraldehyde, or formalin. This can be achieved through standard transcardial perfusion typically used to harvest other organs. It is recommended that shortly after sacrifice the eyes be injected with fixative just under the lens and stored in fixative.
In the present study the subject was deeply sedated with ketamine hydrochloride (10 mg/kg, i.m.), euthanized with an overdose of sodium pentobarbital (25 mg/kg, i.v.) and perfused transcardially with 0.1 M PBS until completely exsanguinated. This is followed by a 4% paraformaldehyde solution in PBS for 5 min (~1 liter).

### Part 2: Removal of the eyeball from the orbital cavity

For easier access to the eyeball it is recommended to first remove the brain. Once the brain has been removed the thin-walled orbit bone is readily apparent. Use the bone rongeurs to slowly chip away the wall of the orbit. Cut away the ocular muscles with a scalpel and remove the connective tissue from the eyeball. Carefully cut the optic nerve, this can be used in electronic microscopy studies. The eyeball should now be released from the orbital cavity.

### Part 3: Dissect the retina from the eyecup

Place the eye into a Petri dish with PBS to keep the retina from drying. A dissecting microscope or table mounted magnifying light stand is useful in the dissection, but not a necessity. Remove the cornea by cutting the sclera closely to the perimeter of the cornea at the level of the ora serrata with a pair of spring scissors and remove the lens with the forceps.
Use a paintbrush, forceps, and spring scissors to remove the retina from the sclera. This is done by separating the retina from the sclera and then cutting the sclera away with the scissors. One must carry out this process in small increments so as not to damage or tear the retinal tissue. The sclera is not readily separated from the remnant optic nerve so carefully cut the sclera around the optic nerve.
At this point the retina has retained its curved shape and needs to be flattened for sampling. Before flattening the retina, the vitreous humour, which has the consistency of jelly, can be removed in a lump. To flatten the retina onto a slide, make several radial cuts with a scalpel blade. The residual vitreous humour can now be removed with ordinary filter paper and a paintbrush. The retinal ganglion cell layer is exposed at this point so it is imperative to be gentle when removing the vitreous humour. If the optic nerve is still attached at the optic disc, remove the optic nerve without ripping the retina using a scalpel blade and a pair of spring scissors. This is now a flat mount retina (Figure 1) and the fovea should be apparent as a dark patch in the temporal/ventral direction from the optic disc.

### Part 4: Sampling

There are a number of options for sampling the retina. Here we will describe the flatmount preparation and isodentric sampling. For both procedures flatmount the retina with the optic fiber layer away from the slide. If the intention is to examine the retinal ganglion cell layer in the flatmount preparation, it is necessary to either remove or bleach the pigmented epithelium. To remove the pigmented epithelium use a paintbrush lightly to remove some of this layer, this tends to damage the photoreceptor layer. Bleaching involves soaking the retina in potassium permanganate solution (0.25%) for 1 hour, washing in distilled water, and clearing in oxalic acid (5%) for 5 minutes.^2^
If the intention is to examine cell distribution and morphology of each layer throughout the retina, then we suggest isometric sampling of the retina. The retina should be flatmounted on a slide and kept moist with PBS. For isometric sampling cut small pieces of tissue equidistant from the optic disc in the nasal, temporal, upper and lower directions (Figure 1). It is not necessary to bleach the pigmented epithelium in this preparation. These pieces can now be sectioned in the coronal plane with a cryostat, vibratome, or ultramicrotome depending on the research question. For cryostat sectioning, the pieces should be cryoprotected in 30% sucrose overnight and frozen in the context of a mounting medium on dry ice. Alternatively, the samples can be embedded in agar and sliced on a vibratome. The cryostat and vibratome preparations will reliably yield sections as thin as 4 m. If thinner sections are required (e.g. for electron microscope preparation), it is necessary to prepare the samples for the ultramicrotome. To do this, the sections should be post-fixed in osmium tetroxide for 1 hour under the fumehood. This is followed by dehydration in a graded ethanol series (50, 70, 95, 95, 100, 100%) and 100% propylene oxide . The tissue is then embedded in Epon (EMBed-812 embedding kit).

### Part 5: Representative Results:

In our laboratory, we routinely perform immunohistochemistry on cryosectioned retinae (Figure 2). In this case we are interested in the isodensity of cannabinoid receptors (CB1) in the primate retina. We also examine the effects of prenatal ethanol exposure on the primate visual system. To this end, we are interested in cell density and layer thickness in the fovea and in the peripheral retina. To accomplish this, we embed pieces of retina in Epon, slice at 700nm on an ultramicrotome, and stain with 1% toluidine blue (Figure 3).


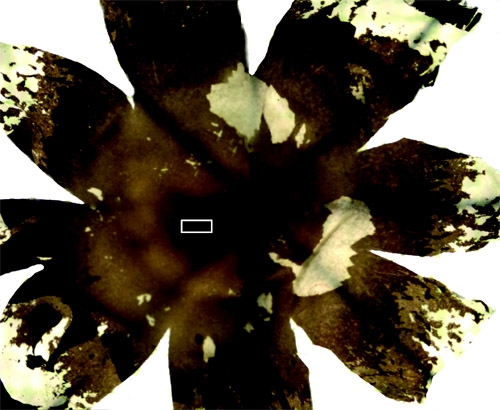
 **Figure 1 Flatmount Retina. **Radial cuts are used to flatten the retina.


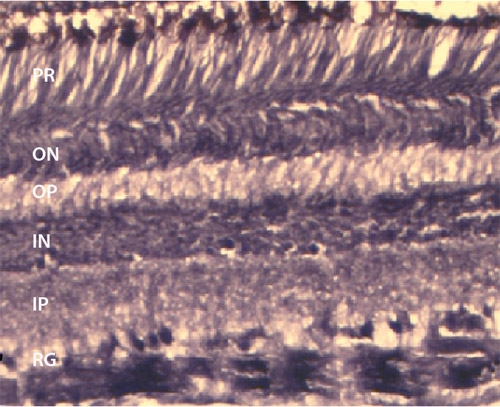
 **Figure 2 Immunostaining. **Cryosection of peripheral retina immunostained for CB1 where the retinal ganglion cells are heavily labeled with some labeling in the inner and outer nuclear layers. The thickness of this section is 14 m and stained on the slide. RG - retinal ganglion layer; IP - inner plexiform layer; IN -  inner nuclear layer; OP -  outer plexiform layer; ON - outer nuclear layer.


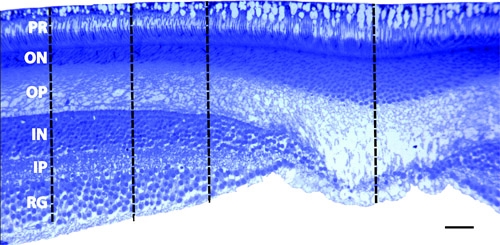
 **Figure 3 Fovea. **Section through the fovea that was embedded in Epon and sliced on an ultramicrotrome at 700nm. The photoreceptor layer (PR) was used to align the sections.  Notice that the entire extent of the photoreceptors can be identified indicating an appropriate angle in the coronal plane.  Density measurements were taken at 300 m, 500 m, and 800 m from the center of the foveal pit using the Bioquant Imaging system. RG - retinal ganglion layer; IP - inner plexiform layer; IN -  inner nuclear layer; OP -  outer plexiform layer; ON - outer nuclear layer; scale bar = 50 m

## Discussion

The preparation of the retina as a wholemount allows for the analysis of the topography and spatial distribution of either the ganglion cell layer or the endothelial cells of the retinal blood vessels^3^. Quantification of cell density in the periphery of primate retina is readily accomplished. however, in perifoveal and foveal regions, the stacking of multiple layers in the ganglion cell layer obstructs quantification. To circumvent this potential bias, the fovea and perifoveal region can be dissected from the wholemount preparation, embedded in Epon, and serially sectioned using an ultramicrotome to obtain semi-thin sections in the coronal plane^2,4^. There are a number of other disadvantages to the wholemount preparation, which can be overcome with alternative sampling paradigms.

Taking isometric samples from the retina and sectioning in the coronal plane on either a cryostat or vibratome allows for specific examination of the different layers, which cannot be readily performed on a wholemount preparation. Sectioning in this manner also allows for the application of multiple immnohistochemistry protocols^5^. These sections can then be removed from the slide, embedded in Epon and sliced on an ultramicrotome. With an ultramicrotome minor changes in the cutting angle can be made to ensure a standard coronal plane through the photoreceptors. Once a standard plane has been obtained, layer thickness can be measured and compared between retinal regions and subjects. Furthermore, Epon embedded tissue can be used in electron microscopy studies to reveal ultrastructural characteristics of the retina^6^.
